# Diffuse membranoproliferative glomerulonephritis with focal sclerosis and renal amyloidosis in an adult male with autosomal dominant dystrophic epidermolysis bullosa: a case report

**DOI:** 10.1080/0886022X.2019.1614056

**Published:** 2019-09-09

**Authors:** Karim M. Soliman, Tibor Fülöp, David W. Ploth, Johann Herberth

**Affiliations:** a Department of Medicine, Division of Nephrology, Medical University of South Carolina, Charleston, SC, USA;; b Department of Medicine, Division of Nephrology, Cairo University, Cairo, Egypt;; c Medical Services, Ralph H. Jonson VA Medical Center, Charleston, SC, USA

**Keywords:** Autosomal dominant dystrophic epidermolysis bullosa, amyloid, glomerulonephritis, focal sclerosis

## Abstract

Previous reports of glomerular disease in adult patients with autosomal dominant dystrophic epidermolysis bullosa (EB) are limited and include post-infectious glomerulonephritis, IgA nephropathy, amyloidosis, and leukocytoclastic vasculitis. To our knowledge, membranoproliferative glomerulonephritis (MPGN) has not been described before. We report a case of a 39-year-old male with autosomal dominant dystrophic EB, presenting with bilateral leg swelling of one-week duration. There was no other significant past medical history. The physical examination was remarkable for scars and erosions over all body areas, with all extremities with blisters and ulcers covered, absent finger and toenails and bilateral lower extremity edema. Serum creatinine was 0.9 mg/dL, albumin 1.3 g/dL and urine protein excretion 3.7 g/24 h. Viral markers (hepatitis-B, C, and HIV), complement c3 and c4 levels and auto-immune antibody profile all remained negative or within normal limits. Renal ultrasound and echocardiogram were normal. Renal biopsy recovered 14 glomeruli, all with proliferation of mesangial and endothelial cells as well as an expansion of the mesangial matrix, focal segmental sclerosis and amorphous homogeneous deposits demonstrating apple-green birefringence under polarized light with Congo red stain. Our observation emphasizes the importance of recognizing MPGN and secondary amyloidosis in patients with EB, especially with the availability of newer treatment modalities.

## Introduction

Epidermolysis bullosa (EB) is a group of genetic conditions that result in painful easy blistering of the skin and mucous membranes secondary to friction or minor trauma [1]. Some types are autosomal recessive while others are autosomal dominant [2]. According to extent and severity of the disease, there are four main types: EB simplex, dystrophic EB, junctional EB, and Kindler syndrome [3]. The diagnosis is suspected based on symptoms and confirmed by skin biopsy or genetic testing [3]. Glomerular diseases in patients with autosomal dominant EB have been reported in case reports; Kagan et al., for instance, described the occurrence of hereditary nephritis and pretibial EB in siblings [4]. A potential association with amyloidosis or multiple myeloma has been reported by several groups [5–7]. Theories that link these diseases have been postulated, but due to low prevalence of co-occurrence, causative relationships have not yet been conclusively established. It is interesting to note that novel therapies in the treatment of EB utilize compounds with utility in the treatment of glomerular diseases, e.g., mycophenolate mofetil was recently reported as a potential therapeutic agent for EB dystrophica in patients intolerant of cyclosporine [8]. Since EB can complicate vascular access placement and the above reported glomerular diseases are potentially amenable to therapy, it is imperative that clinical practitioners are aware of these disease associations and consider therapeutic interventions early [9–11]. To our knowledge, we are reporting the first known association of EB and membranoproliferative glomerulonephritis (MPGN).

## Case presentation

A 39-year-old male with past medical history significant only for autosomal dominant dystrophic EB characterized by recurrent blisters and erosions on his whole body and oral mucosa since birth presented with complaints of generalized weakness, inflammatory/bullous changes and swelling of legs of several weeks’ duration. There were no associated symptoms of abdominal pain, hematuria, oliguria, arthralgia, oro-genital ulcers, photosensitivity, cough, hemoptysis, palpitations or shortness of breath. The patient was a nonsmoker and had no history of alcohol or drug abuse.

**Figure 1.**
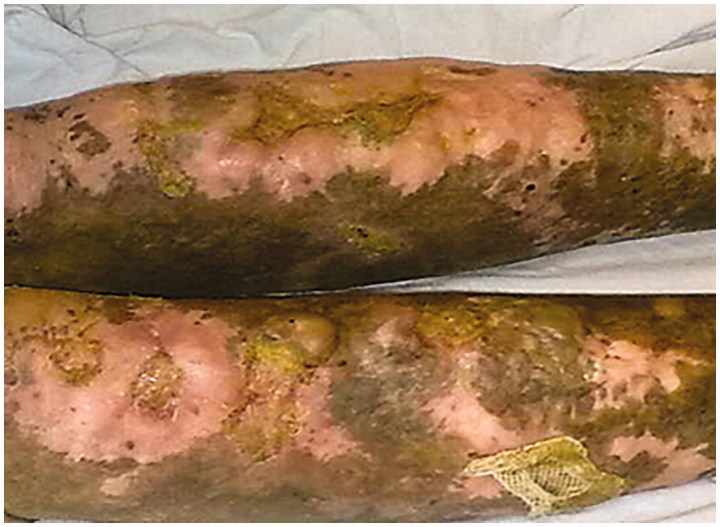
Blisters and ulcers were observed over both lower extremities with moderate pitting edemas.

**Figure 2.**
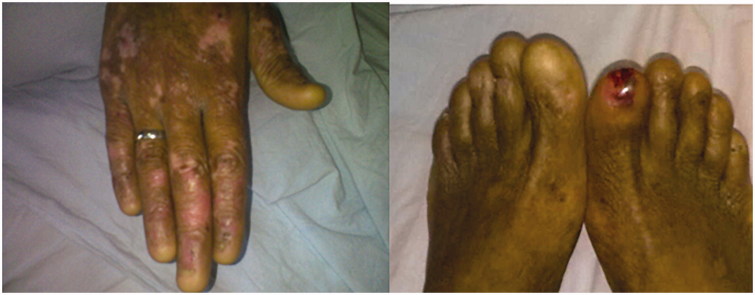
Absent finger and toe nails.

**Figure 3.**
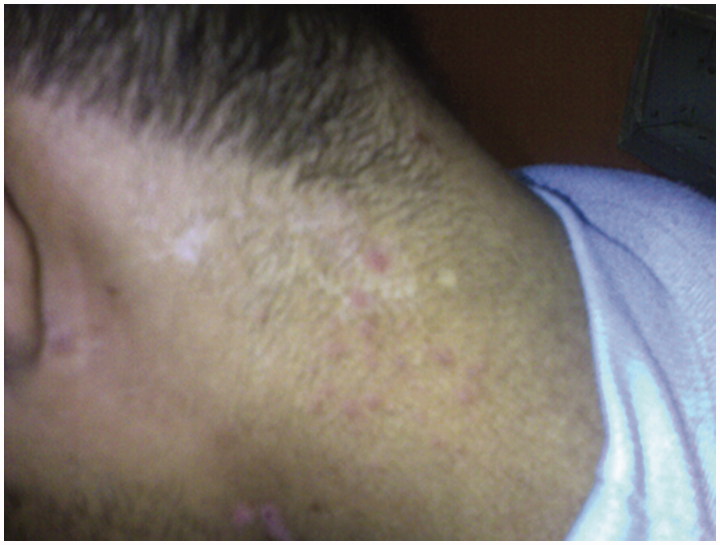
Scattered areas of cicatricial alopecia on the scalp.

**Figure 4.**
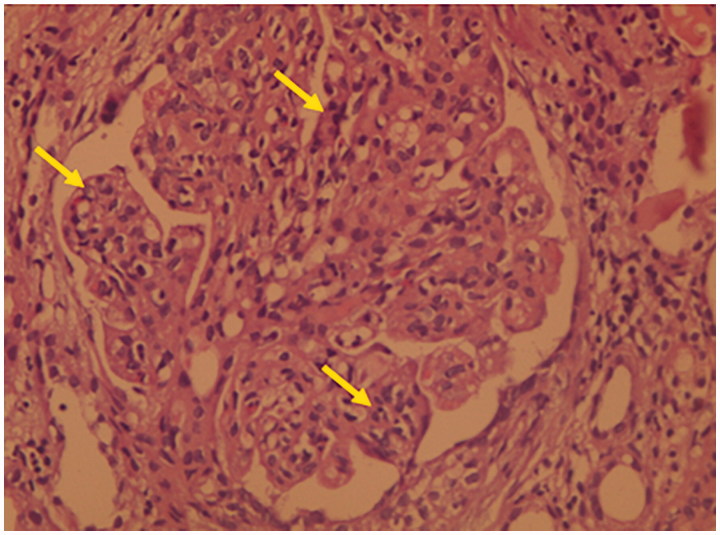
Hematoxylin and eosin stains showing proliferation of mesangial and endothelial cells and an expansion of the mesangial matrix.

**Figure 5.**
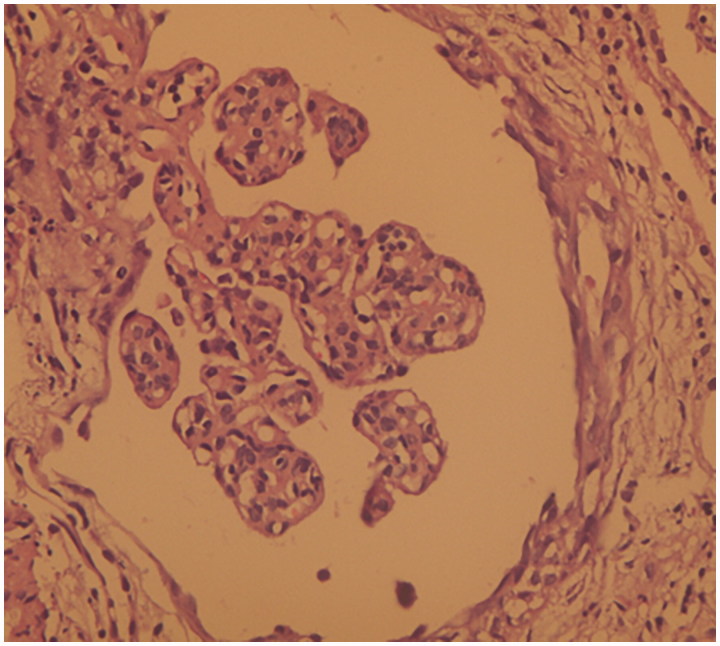
Hematoxylin and eosin stains demonstrating focal segmental sclerosis.

Vitals signs revealed a blood pressure of 110/70 mmHg, a heart rate of 76 beats per minute (regular), a respiratory rate of 16/min and an axillary temperature of 37.4 °C. Oxygen saturation was 98% while breathing on ambient air. His physical examination was remarkable for visible scars and erosions with areas of hyper- and hypopigmentation over the face, neck, chest, abdomen, back and all extremities. Blisters and ulcers were noted over both lower extremities with moderate pitting edemas (Figure 1) and absent finger and toe nails (Figure 2). Scattered areas of cicatricial alopecia on the scalp were also noted (Figure 3). Otherwise, neurological, respiratory and cardiovascular examinations were normal, without organomegaly or any signs of chronic liver disease or heart failure. Laboratory investigations showed normocytic and normochromic anemia with hemoglobin 9.8 g/dL, corrected serum calcium 9.1 mg/dL, serum creatinine 0.9 mg/dL, aspartate aminotransferase 15 U/L (normal range: 5–34 U/L), alanine aminotransferase 16 U/L (normal range: 5–45 U/L), bilirubin 0.5 mg/dL, prothrombin time international normalized ratio (INR) 1.0, serum albumin 1.3 g/dL, cholesterol 270 mg/dL, and erythrocyte sedimentation rate 120 mm/h. Urinalysis was bland with no active sediment, however with 3+ protein and proteinuria of 3.7 g/24 h on timed specimen. Interestingly, despite mild nephrotic range proteinuria, albumin was disproportionally low, probably due to loss from skin oozing as well as a negative acute phase reactant to the chronic inflammatory state. Our differential diagnosis at this time was limited to minimal change disease, membranous nephropathy, focal segmental glomerulosclerosis, or amyloid. Absence of active urinary sediment and the clinical presentation of nephrotic syndrome made us rule out any glomerular proliferative disorder. Urine culture remained without growth. Hepatitis B surface antigen, hepatitis C antibody, and HIV antibody were negative. Test results for antinuclear antibodies, anti-double stranded antinuclear antibodies, anti-neutrophilic cytoplasmic antibodies, cryoglobulin, and C3 as well as C4 came back negative or within normal range. Renal ultrasound showed the right kidney measuring 13.7 × 7.5 cm and the left 14.7 × 7.3 cm with good corticomedullary differentiation and without hydronephrosis, masses, or nephrolithiasis. Echocardiogram was normal. Dermatological consultation confirmed the diagnosis of autosomal dominant dystrophic EB with overlying chronic infection. Subsequently, a percutaneous ultrasound-assisted renal biopsy recovered a tissue specimen with 14 non-hyalinized glomeruli, all with proliferation of mesangial and endothelial cells and an expansion of the mesangial matrix (arrows in Figure 4), focal segmental sclerosis (Figure 5), and amorphous homogeneous eosinophilic deposits which displayed an apple-green birefringence under polarized light after Congo red staining pathognomonic for amyloid deposition. The diagnosis of diffuse MPGN with focal sclerosis and likely secondary amyloid deposition was made. The patient was conservatively placed on angiotensin converting enzyme inhibitor and colchicine [12,13].

## Discussion

Four main patterns of glomerular diseases have been described in EB. Streptococcal infection of the skin is a common complication in patients with EB, placing them at a higher risk of post-infectious glomerulonephritis [14–16]. Mesangial IgA nephropathy has also been reported and is thought to be secondary to repeated mucocutaneous infections [14,17]. Clinically, these patients present with hematuria, hypertension, mild proteinuria, and deteriorating renal function. The third entity reported in EB patients is secondary renal amyloidosis. Unlike the previously mentioned glomerular diseases, nephrotic syndrome is a characteristic feature in amyloidosis [5,7,18–21]. In patients with severe generalized forms of EB, elevated serum amyloid A protein levels may be observed without evidence of renal amyloidosis but the significance of this finding is unclear [19]. All three forms of renal parenchymal diseases may progress to chronic renal insufficiency even if the causes are treatable. This is likely related to chronic and repeated injuries of the renal parenchyma secondary to repeated cycles of inflammation and infection of the skin. Therefore, efforts to reduce the latter may be beneficial in reducing the risks of renal impairment. This, however, may be a difficult option in patients with severe forms of autosomal dominant EB due to genetic predisposition. Lastly, leukocytoclastic vasculitis is the fourth entity of renal involvement [22]. To our knowledge, MPGN with focal sclerosis and renal amyloidosis as a potential complication of EB has not been reported before. Focal sclerosis could be secondary to the healing process of MPGN. The prevalence of secondary amyloidosis in autosomal dominant dystrophic EB may be underappreciated. The fragility of the skin and its susceptibility to infections may render organ biopsy more difficult and riskier. Moreover, clinical features of secondary amyloidosis are often nonspecific and subcutaneous swellings along with tissue congestions may be clinically mistaken for other causes of edema, particularly that of the renal type. An early control of the inflammatory processes can be postulated to prevent the emergence of secondary amyloid. In our case, as in many other complex cases before, renal biopsy has proven to be indispensable to secure the diagnosis. In the modern era, such interventions can easily be accomplished with US guidance and with a low rate of complications even in training facilities [23,24]; hence, with the emergence of new treatment modalities for amyloidosis, we recommend physicians to consider the possibility of secondary amyloidosis for patients affected with EB. Several treatment options are still under investigation and not yet generally approved. Bone marrow transplant has been proposed to cause cure in patients with EB, however with several failed attempts [25]. Sulforaphane, a compound found in broccoli, was found to reduce blistering in a mouse model [26]. Systemic granulocyte-colony stimulating factor (G-CSF) may promote increased wound healing in people with dystrophic EB [27]. Transplanting skin derived from genetically modified stem cells onto the wound surfaces has been proposed to show improvements [28].

Important limitations of our paper are the lack of immunofluorescence and electron microscopic studies and dedicated evaluation for amyloid-A deposition with pronase digestion and subsequent evaluation for presence of AA amyloid. Accordingly, while the focus of this paper was not the presence of amyloid, the diagnosis of MPGN remains only presumptive and needs further studies to corroborate these findings. Moreover, the relative contribution of amyloid vs. MPGN to proteinuria and clinical nephrosis in our case study remained insufficiently defined. Our report focuses on the novel finding of MPGN in a patient with EB. Monoclonal gammopathy of renal significance was described in association with MPGN and secondary renal amyloidosis is a known entity associated with EB [29]. The finding of apple-green birefringence after Congo red staining in the glomerular deposits in our case suggests a possible association of amyloidosis with the histopathologic findings in the glomeruli [29–31]. While a potential association of secondary amyloidosis because of chronic inflammatory states in EB patients appears plausible, further studies have to determine the prevalence of primary versus secondary amyloidosis in this vulnerable population.

## Conclusions

We report a case of a patient with autosomal dominant dystrophic EB developing progressive bilateral lower extremity swellings and nephrotic-range proteinuria. Renal biopsy disclosed diffuse MPGN with focal sclerosis and renal amyloid deposition. Membranoproliferative glomerulonephritis may represent a novel renal association for dystrophic EB.
